# Autoimmune hepatitis and primary biliary cholangitis overlap syndrome after COVID-19

**DOI:** 10.4322/acr.2023.422

**Published:** 2023-03-10

**Authors:** Marlone Cunha-Silva, Eloy Vianey Carvalho de França, Raquel Dias Greca, Daniel Ferraz de Campos Mazo, Larissa Bastos Eloy da Costa, Priscilla Brito Sena de Moraes, Clauber Teles Veiga, Guilherme Rossi Assis-Mendonça, Ilka de Fátima Santana Ferreira Boin, Raquel Silveira Bello Stucchi, Tiago Sevá-Pereira

**Affiliations:** 1 Universidade Estadual de Campinas (UNICAMP), School of Medical Sciences, Centro de Diagnóstico de Doenças do Aparelho Digestivo (GASTROCENTRO), Department of Internal Medicine, Division of Gastroenterology, Campinas, SP, Brasil; 2 Universidade Estadual de Campinas (UNICAMP), School of Medical Sciences, Department of Pathology, Campinas, SP, Brasil; 3 Universidade Estadual de Campinas (UNICAMP), School of Medical Sciences, Department of Surgery, Campinas, SP, Brasil; 4 Universidade Estadual de Campinas (UNICAMP), School of Medical Sciences, Department of Internal Medicine, Division of Infectious Diseases, Campinas, SP, Brasil

**Keywords:** COVID-19, Hepatitis, Autoimmune, Liver Diseases, SARS-CoV-2

## Abstract

COVID-19 is commonly associated with high serum levels of pro-inflammatory cytokines, and the post-infection status can disturb self-tolerance and trigger autoimmune responses. We are reporting a 45-year-old male who was admitted with fatigue, jaundice, elevated liver enzymes (with cholestatic pattern), and acute kidney injury two weeks after recovering from a mild SARS-CoV-2 infection. Serologies for viral hepatitis and anti-mitochondrial antibody were negative, while anti-nuclear and anti-smooth muscle antibodies were positive. There were no signs of chronic liver disease, and a magnetic resonance cholangiography showed no dilatation of biliary ducts. Histologic evaluation of the liver evidenced numerous foci of lobular necrosis without ductopenia or portal biliary reaction. Considering the autoantibody profile and histologic changes, the medical team started oral prednisone, but there was a suboptimal biochemical response in the outpatient follow-up. Two months later, a second liver biopsy was performed and revealed non-suppurative destructive chronic cholangitis, extensive areas of confluent necrosis with hepatocytes regenerating into pseudorosettes, and numerous plasma cells. According to the Paris Criteria, the patient was then diagnosed with an autoimmune hepatitis-primary biliary cholangitis overlap syndrome (AIH-PBC-OS). After adding azathioprine and ursodeoxycholic acid to the treatment, there was a satisfactory response. This is the second worldwide report of an AIH-PBC-OS triggered by COVID-19, but the first case with a negative anti-mitochondrial antibody. In this setting, histologic evaluation of the liver by an experienced pathologist is a hallmark of achieving the diagnosis and correctly treat the patient.

## INTRODUCTION

SARS-CoV-2 (severe acute respiratory syndrome coronavirus 2) infection is commonly associated with high serum levels of pro-inflammatory cytokines (particularly interleukins, tumor necrosis factor, granulocyte-macrophage colony-stimulating factor), which leads to hyperstimulation of the immune system.^1^ The post-infection status can disturb self-tolerance and trigger autoimmune responses through cross-reactivity with host cells.^2^ Therefore, patients may present with autoimmune manifestation months after the acute viral infection, such as Guillain-Barré syndrome, vasculitis, thyroiditis, hemolytic anemia, immune thrombocytopenic purpura, autoimmune hepatitis, and several other entities.^1^^,^^3^

Regarding the liver, the definitive diagnosis of autoimmune damage is challenging as other aspects may be involved, such as drug-, virus-, vaccination-induced injuries, or even multifactorial. ^4^^-^^6^ Thus, histologic analysis of the liver by an experienced pathologist is quite helpful. We aim to report a case of a man with acute cholestatic hepatitis right after COVID-19, which we considered the triggering event. This patient was subsequently diagnosed with autoimmune hepatitis-primary biliary cholangitis overlap syndrome (AIH-PBC-OS).

## CASE REPORT

A 45-year-old man presented with fever, cough, and body aches and was diagnosed with SARS-CoV-2 infection after testing with RT-PCR (reverse transcriptase-polymerase chain reaction). The illness was not severe, did not require hospitalization, and there was a full recovery after taking 1 tablet of loratadine (10mg), acetaminophen (500mg), and azithromycin (500mg) daily for 5 days. Two weeks later, he complained of weakness, jaundice, and pruritus, with no fever. He denied recent trips, consumption of alcohol, teas, herbal products, or use of illicit drugs. On physical examination, there was jaundice with no signs of chronic liver disease or enlarged lymph nodes.

Laboratory tests showed high serum levels of liver enzymes (acute cholestatic hepatitis), total/direct bilirubin, and ferritin, with no changes in the international normalized ratio (INR), albumin, and gamma-globulin, as presented in Table 1.

**Table 1:** Initial laboratory tests of the reported patient

**Test**	**Result**	**RR**	**Test**	**Result**
**Hemoglobin (g/dL)**	7.0	14.0 - 18.0	**Anti-HAV IgM**	**-**
**Leucocytes x10^3^ (/mm^3^)**	7.61	4.0 - 10.0	**Anti-HAV IgG**	**+**
**Platelets x10^3^ (/mm^3^)**	310	150 - 400	**HBsAg**	**-**
**AST (IU/L)**	70	< 50	**Anti-HBc IgG**	**-**
**ALT (IU/L)**	156	< 50	**Anti-HBs**	**-**
**ALP (IU/L)**	250	< 129	**Anti-HCV**	**-**
**GGT (IU/L)**	609	< 60	**Anti-HIV**	**-**
**Total bilirubin (mg/dL)**	28.5	< 1.2	**ANA**	**+ (1:1280)**
**Direct bilirubin (mg/dL)**	21.0	< 0.2	**ASMA**	**+ (1:160)**
**Albumin (g/dL)**	3.5	3.5 - 5.2	**AMA**	**-**
**Gamma-globulin (g/dL)**	1.5	0.64 - 2.10	**Anti-LKM1**	**-**
**Urea (mg/dL)**	312	17 - 43	**Anti-dsDNA**	**-**
**Creatinine (mg/dL)**	3.2	0.7 - 1.2	**Anti-Ro**	**-**
**Ceruloplasmin (mg/dL)**	29	20 - 60	**Anti-La**	**-**
**Ferritin (ng/mL)**	7,059	30 - 400	**CMV IgM**	**-**
**TSI**	39%	20 - 50%	**CMV IgG**	**+**
**A1AT (mg/dL)**	154	90 - 200	**EBV IgM**	**-**
**IgG (mg/dL)**	1,509	952 - 1,538	**EBV IgG**	**+**
**IgM (mg/dL)**	108	73 - 171	**Toxoplasmosis IgM**	**-**
**INR**	1.2	< 1.25	**Toxoplasmosis IgG**	**+**

Laboratory tests collected at hospital admission showed non-severe acute cholestatic hepatitis, elevated serum levels of bilirubin and ferritin, impaired renal function, negative viral serologies, positive ASMA and ANA, but normal gamma-globulins. ALP: alkaline phosphatase; ALT: alanine aminotransferase; AMA: anti-mitochondrial antibody; ANA: anti-nuclear antibody (nuclear fine speckled); Anti-dsDNA: anti-double stranded deoxyribonucleic acid antibody; Anti-LKM1: anti-liver kidney microsome-1 antibody; ASMA: anti-smooth muscle antibody; AST: aspartate aminotransferase; A1AT: alpha-1 antitrypsin; CMV: cytomegalovirus; EBV: Epstein-Barr virus; GGT: gamma-glutamyl transferase; HAV: hepatitis A virus; HBs/HBc: hepatitis B virus serological tests; HCV: hepatitis C virus; HIV: human immunodeficiency virus; Ig: immunoglobulin; INR: international normalized ratio; TSI: transferrin saturation index.

He also had impaired renal function with normal serum levels of complements, creatine kinase, and no changes on urinalysis. Serologies for viral hepatitis, HIV, cytomegalovirus, Epstein-Barr virus, and toxoplasmosis were unremarkable, while anti-nuclear antibody (ANA) and anti-smooth muscle antibody (ASMA) were positive (1:1280 nuclear fine speckled pattern and 1:160, respectively). In contrast, anti-mitochondrial antibody (AMA), anti-double stranded deoxyribonucleic acid antibody (anti-dsDNA), and anti-liver kidney microsome-1 antibody (anti-LKM1) were both negative (Table 1). A new RT-PCR-SARS-CoV-2 was negative.

**Figure 1:**
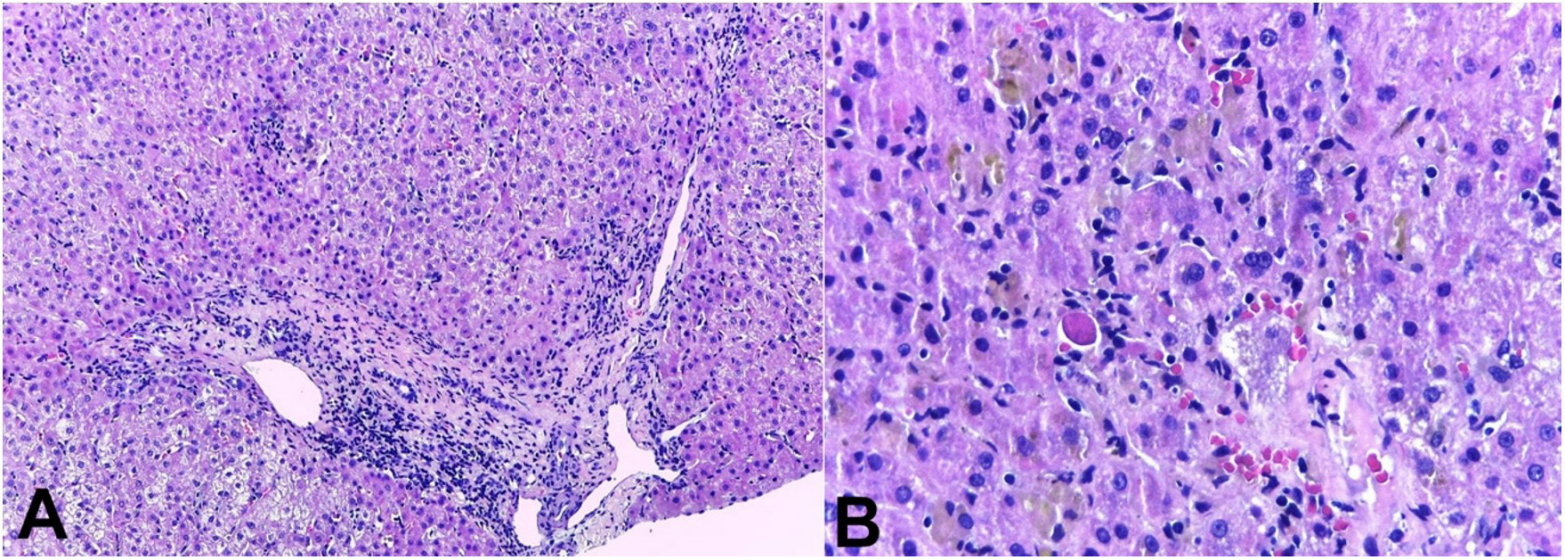
Photomicrographs of the liver biopsy. **A** - acute/subacute pattern of injury: there is a lymphocyte-predominant portal and lobular inflammation with spotty hepatocellular necrosis without significant ductal aggression (H&E, 10x); **B** - the “cholestatic hepatitis” injury with spotty necrosis, and acidophil bodies (H&E, 20x).

The liver had a finely heterogeneous echotexture, thin borders, and a regular surface on abdominal ultrasound. Magnetic resonance cholangiography showed normal biliary ducts. Renal function exams normalized without the need for dialysis. In the lack of a specific diagnosis, a real-time ultrasound-guided percutaneous liver biopsy was performed, and the histologic evaluation showed acute/subacute hepatitis, with numerous foci of lobular necrosis, associated with moderate canalicular and intrahepatocytic cholestasis, predominantly in zone 3, without ductopenia or portal biliary reaction (Figures 1A and 1B).

**Figure 2:**
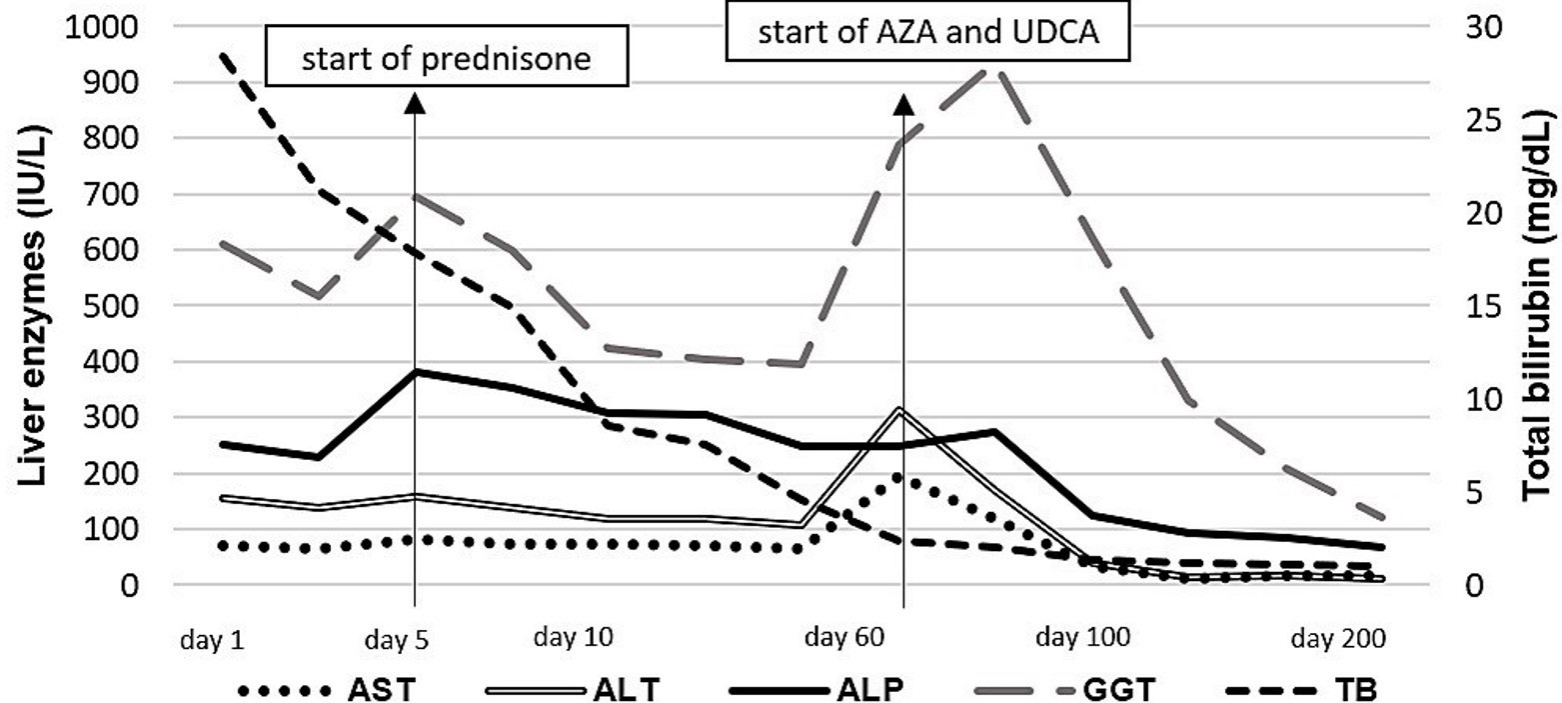
Laboratory tests over time. ALP: alkaline phosphatase; ALT: alanine aminotransferase; AST: aspartate aminotransferase; AZA: azathioprine; GGT: gamma-glutamyl transferase; TB: total bilirubin; UDCA: ursodeoxycholic acid.

A new increase in serum levels of liver enzymes was observed, so the medical team decided to start oral prednisone 40mg/day for 7 days followed by symptoms improvement. The dosage was reduced to 30mg/day for the next 7 days and maintained 20mg/day in the outpatient follow-up. The biochemical response was suboptimal (Figure 2), and a second liver biopsy was then performed two months later.

**Figure 3:**
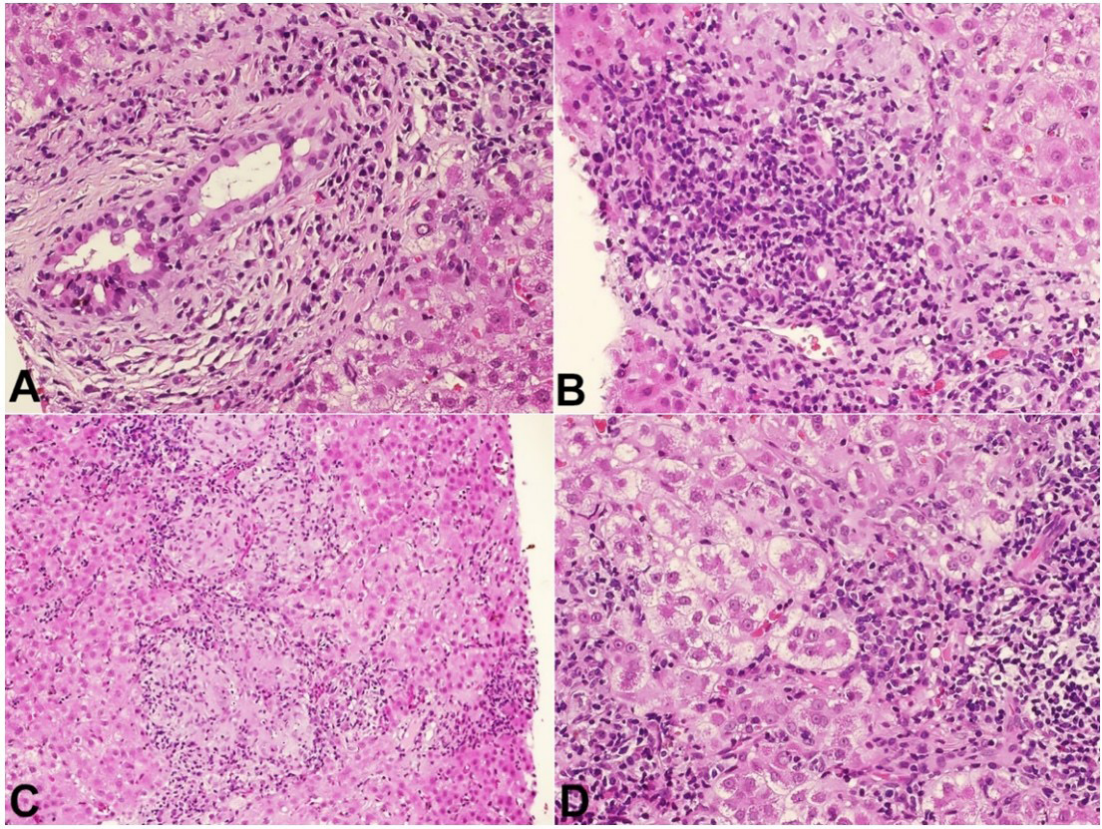
Photomicrograph of the second liver biopsy. **A** - extensive aggression of the bile duct, with cytoplasmic vacuolization, pyknotic nuclei and a plasma-cell rich infiltrate (H&E, 20x); **B** - there is a chronic destructive aggression of the bile duct, with numerous plasma cells and interface activity; **C** - portal and lobular granulomas are also present (H&E, 10x); **D** - confluent necrosis and pseudorosette hepatocellular regeneration (H&E, 20x).

Liver histologic analysis revealed non-suppurative destructive chronic cholangitis, and extensive areas of confluent necrosis with hepatocytes regenerating into pseudorosettes and numerous plasma cells (Figures 3A to 3D).

According to the Paris Criteria^7^ (Table 2), the patient was diagnosed with AIH-PBC-OS, so azathioprine and ursodeoxycholic acid (UDCA) were added to the therapeutic regimen.

**Table 2:** The Paris Criteria for the diagnosis of AIH-PBC-OS^7^

**AIH**	1. ALT ≥ 5x ULN*
	2. IgG ≥ 2x ULN or positive-ASMA*
	3. Liver biopsy: moderate/severe periportal/periseptal lymphocytic necrosis*
**PBC**	1. ALP ≥ 2x ULN or GGT ≥ 5x ULN*
	2. Positive-AMA
	3. Liver biopsy: florid bile duct lesions^*^

At least 2 of 3 accepted criteria for PBC and AIH, respectively, should be present. Histologic evidence of moderate to severe lymphocytic piecemeal necrosis (interface hepatitis) is mandatory for the diagnosis. AIH-PBC-OS: autoimmune hepatitis-primary biliary cholangitis overlap syndrome; AIH: autoimmune hepatitis; ALP: alkaline phosphatase; ALT: alanine aminotransferase; AMA: anti-mitochondrial antibody; ASMA: anti-smooth muscle antibody; GGT: gamma-glutamyl transferase; IgG: immunoglobulin G; PBC: primary biliary cholangitis; ULN: upper limit of normal; *Our patient had 3 positive features for AIH and 2 for PBC.

There was a progressive decrease in serum levels of liver enzymes and bilirubins, which were normal within 7 months of combined therapy. The patient is on azathioprine (1.4 mg/kg/day), UDCA (14 mg/kg/day), and prednisone 5 mg/day with biochemical remission for 24 months. The evolution of liver tests according to the treatment is displayed in Figure 2.

## DISCUSSION

In the setting of SARS-CoV-2 infection with multiple drug administration and hepatic test alterations, virus-induced liver damage and drug-induced liver injury (DILI) must always be considered in the differential diagnosis.^8^ Azithromycin-induced liver injury could justify the initial clinical and liver biochemical abnormalities in the present case. This antibiotic is a well-known but rare cause of clinically apparent liver injury.^9^ It is typically associated with cholestatic hepatitis;^10^ however, acute hepatocellular injury^11^ and even chronic liver disease have been reported, but positive autoantibodies are uncommon.^9^^,^^12^^,^^13^ Loratadine is a rare cause of liver injury, and acetaminophen leads to severe cases of liver toxicity, especially acute liver failure. Still, this injury is dose-dependent, and the amount ingested by the patient was minimal. ^9^ The patient’s clinical and laboratory course associated with the findings of the second liver biopsy made this initial DILI hypothesis less likely.

The association of PBC and AIH is rare, occurring in 1-3% of patients with PBC and 7% of patients with AIH.^14^ The diagnosis is usually a challenge, and the Paris Criteria are the most widely chosen tool.^7^^,^^14^^-^^16^ Many patients are initially diagnosed with PBC and treated with UDCA, but subsequent raises in aminotransferases and gamma-globulin, as well as a poor response to therapy, lead the physicians to suspect an AIH-PBC-OS and change the treatment or perform a liver biopsy.^14^ Although our patient presented with a cholestatic pattern of liver injury, our team decided to start prednisone considering the autoantibodies profile and numerous foci of lobular necrosis, with no relevant biliary changes in the first histologic evaluation of the liver. After the second biopsy, he met the Paris Criteria,^7^ and we promptly added UDCA and azathioprine to the treatment.

A Chinese study^17^ evaluated 74 patients with AIH-PBC-OS and 40 (54%) had negative-AMA. There was no difference in clinical manifestations and major liver function indexes between the negative and positive groups, but the level of immunoglobulin M was significantly lower in patients with negative-AMA (as in our case) compared to those with positive-AMA. Also, there were no significant differences in inflammation and fibrosis stages between the two groups, but the bile duct injury was more significant in the negative than the positive group.

Infections, drugs, environmental factors, or even other diseases may trigger the onset of clinical and laboratory features in patients with AIH-PBC-OS,^16^^,^^17^ and it is already known that the post-COVID-19 status can trigger autoimmune disorders by antigenic mimicry between viral and human proteins.^2^ This may have occurred in our case since he had positive ANA and ASMA.

Liver injury in SARS-CoV-2 seems to be multifactorial. The suggested mechanisms include direct cytotoxicity from active viral replication in the liver, immune-mediated damage, hypoxic change-induced respiratory failure, vascular changes due to coagulopathy, and drug- or vaccination-induced liver injury.^4^^-^^6^^,^^18^ COVID-19-related cholangiopathies have already been reported, such as PBC alone,^19^ or sclerosis cholangitis alone (which is usually more severe and has a higher need for liver transplantation).^4^^,^^20^^,^^21^ We found some reports of AIH following COVID-19;^3^^,^^22^^,^^23^ however, only one case of AIH-PBC-OS triggered by SARS-CoV-2 has been published worldwide. It was a 57-year-old male with positive AMA, ASMA, and Anti-dsDNA.^24^ As in our case, high serum levels of gamma-glutamyl transferase and ferritin were found. In contrast, our patient had normal gamma-globulin, with higher serum levels of total and direct bilirubin. None of them had dilatation of the biliary ducts on magnetic resonance cholangiography.^24^ Table 3 shows the comparison between the main features of these cases.

**Table 3:** Comparison of the main features between our patient and that reported by Singh et al.^24^

**Features**	**Our patient**	**Singh´s case^24^**
**Gender / age**	male / 45y	male / 57y
**AST (IU/L)**	70	371
**ALT (IU/L)**	156	246
**ALP (IU/L)**	250	29
**GGT (IU/L)**	609	655
**Total bilirubin (mg/dL)**	28.5	2.1
**Gamma-globulin (g/dL)**	1.5	3.8
**IgG (mg/dL)**	1,509	4,049
**IgM (mg/dL)**	108	281
**Ferritin (ng/mL)**	7,059	3,275
**TSI**	39%	86%
**ANA**	positive	negative
**ASMA**	positive	positive
**AMA**	negative	positive
**Anti-dsDNA**	negative	positive
**Anti-LKM1**	negative	negative

ALP: alkaline phosphatase; ALT: alanine aminotransferase; AMA: anti-mitochondrial antibody; ANA: anti-nuclear antibody; Anti-dsDNA: anti-double stranded deoxyribonucleic acid antibody; Anti-LKM1: anti-liver kidney microsome-1 antibody; ASMA: anti-smooth muscle antibody; AST: aspartate aminotransferase; GGT: gamma-glutamyl transferase; Ig: immunoglobulin; TSI: transferrin saturation index.

The best therapeutic approach to AIH-PBC-OS seems to be the combination of UDCA plus corticosteroids with or without azathioprine, but some studies have shown a better response to treatment aimed at AIH or PBC alone. Physicians may target therapy at the major inflammatory activity (hepatocellular or cholestatic), further evaluating the need to add other drugs.^14^^,^^15^ Cholestyramine or rifampicin may be needed to control pruritus.^7^

The prognosis of patients with AIH-PBC-OS seems to be worse compared to those with AIH or PBC alone. Progression to cirrhosis is more common and the need for liver transplantation is higher in subjects with overlap syndrome.^14^^,^^24^^,^^25^ A cohort study^26^ involving 135 PBC patients (26 with AIH-PBC-OS) showed that liver transplantation or death occurred in 38% of patients with AIH-PBC-OS versus 19% of those with PBC alone during a six-year mean follow-up period.

Our patient had a significant increase in serum urea and creatinine levels when he was admitted to the hospital, but an improvement was seen with no need for dialysis. We suspected an immune-mediated mechanism or hemophagocytic syndrome;^27^ however, the investigation was unremarkable, and the true etiology of renal damage was unclear.

## CONCLUSION

The post-COVID-19 status has been associated with autoimmune phenomena. Although we cannot be certain that this occurred in our patient, this is the main hypothesis considering the literature data. Physicians should suspect AIH-PBC-OS after SARS-CoV-2 infection, even in the setting of negative-AMA. Histologic evaluation of the liver plays an essential role in this diagnosis.
